# 4-Amino-*N*-(6-chloro-5-methoxy­pyrimidin-4-yl)benzene­sulfonamide

**DOI:** 10.1107/S1600536810001121

**Published:** 2010-01-16

**Authors:** Hoong-Kun Fun, Jia Hao Goh, C. S. Chidan Kumar, H. S. Yathirajan, B. Narayana

**Affiliations:** a X-ray Crystallography Unit, School of Physics, Universiti Sains Malaysia, 11800 USM, Penang, Malaysia; b Department of Studies in Chemistry, University of Mysore, Manasagangotri, Mysore 570 006, India; c Department of Studies in Chemistry, Mangalore University, Mangalagangotri, Mangalore 574 199, India

## Abstract

In the title compound, C_11_H_11_ClN_4_O_3_S, the S atom is bonded in a distorted tetra­hedral geometry, by two O atoms, a C atom of the benzene ring and an amino N atom. The essentially planar pyrimidine ring [maximum deviation = 0.020 (1) Å] forms a dihedral angle of 87.57 (5)° with the benzene ring. In the crystal structure, pairs of mol­ecules are linked by inter­molecular N—H⋯O hydrogen bonds to generate centrosymmetric *R*_2_^2^(8) ring motifs. In addition, mol­ecules are linked into a three-dimensional extended network by inter­molecular N—H⋯N, N—H⋯O and C—H⋯O hydrogen bonds.

## Related literature

For general background to and applications of the title compound, see: Amir *et al.* (2007 ▶); Calabresi *et al.* (1975 ▶); El-Hashash *et al.* (1993 ▶); Nagaraja *et al.* (2003 ▶); Townsend & Drach (2002 ▶). For a related structure, see: Chohan *et al.* (2008 ▶). For details of hydrogen-bond motifs, see: Bernstein *et al.* (1995 ▶). For the stability of the temperature controller used for the data collection, see: Cosier & Glazer (1986 ▶). For bond-length data, see: Allen *et al.* (1987 ▶).
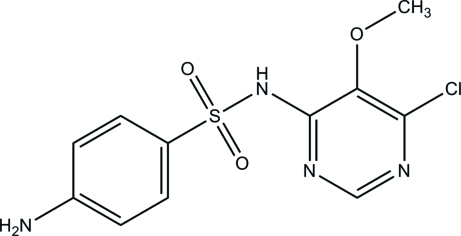


## Experimental

### 

#### Crystal data

C_11_H_11_ClN_4_O_3_S
*M* *_r_* = 314.75
Monoclinic, 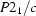

*a* = 12.8792 (6) Å
*b* = 13.3557 (6) Å
*c* = 8.0867 (4) Å
β = 102.396 (1)°
*V* = 1358.57 (11) Å^3^
*Z* = 4
Mo *K*α radiation
μ = 0.45 mm^−1^
*T* = 100 K
0.44 × 0.33 × 0.12 mm

#### Data collection

Bruker SMART APEX DUO area-detector diffractometer
Absorption correction: multi-scan (*SADABS*; Bruker, 2009 ▶) *T*_min_ = 0.829, *T*_max_ = 0.950
20122 measured reflections
4863 independent reflections
4292 reflections with *I* > 2σ(*I*)
*R*_int_ = 0.026

#### Refinement

*R*[*F* ^2^ > 2σ(*F* ^2^)] = 0.030
*wR*(*F* ^2^) = 0.089
*S* = 1.04
4863 reflections
225 parameters
All H-atom parameters refined
Δρ_max_ = 0.53 e Å^−3^
Δρ_min_ = −0.37 e Å^−3^

### 

Data collection: *APEX2* (Bruker, 2009 ▶); cell refinement: *SAINT* (Bruker, 2009 ▶); data reduction: *SAINT*; program(s) used to solve structure: *SHELXTL* (Sheldrick, 2008 ▶); program(s) used to refine structure: *SHELXTL*; molecular graphics: *SHELXTL*; software used to prepare material for publication: *SHELXTL* and *PLATON* (Spek, 2009 ▶).

## Supplementary Material

Crystal structure: contains datablocks global, I. DOI: 10.1107/S1600536810001121/lh2975sup1.cif

Structure factors: contains datablocks I. DOI: 10.1107/S1600536810001121/lh2975Isup2.hkl

Additional supplementary materials:  crystallographic information; 3D view; checkCIF report

## Figures and Tables

**Table 1:** Hydrogen-bond geometry (Å, °)

*D*—H⋯*A*	*D*—H	H⋯*A*	*D*⋯*A*	*D*—H⋯*A*
N1—H1*N*1⋯N4^i^	0.875 (19)	2.616 (18)	3.4230 (14)	153.8 (15)
N1—H2*N*1⋯O1^ii^	0.882 (18)	2.533 (19)	3.3274 (13)	150.2 (15)
N2—H1*N*2⋯O2^iii^	0.880 (18)	2.031 (18)	2.8866 (12)	163.7 (16)
C4—H4*A*⋯O1^ii^	0.944 (16)	2.460 (16)	3.2603 (13)	142.5 (13)

Symmetry codes: (i) 
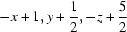
; (ii) 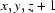; (iii) 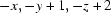.

**Fig. 1.**
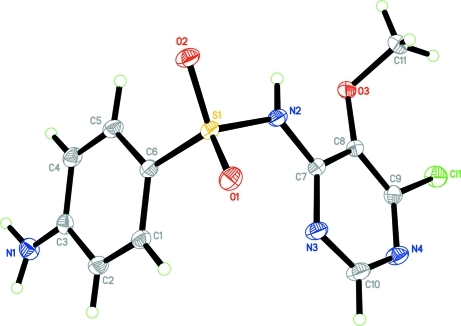
The molecular structure of the title compound, showing 50% probability displacement ellipsoids for non-H atoms and the atom-numbering scheme.

**Fig. 2.**
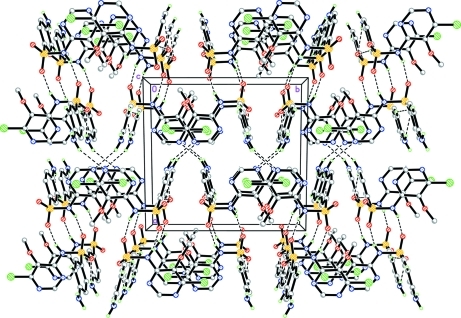
Part of the crystal structure of the title compound, viewed along the c axis, showing a three-dimensional extended network. H atoms not involved in intermolecular hydrogen bonds (dashed lines) have been omitted for clarity.

## supplementary crystallographic information

### Comment

The importance of pyrimidines and analogous compounds in pharmaceutical and biological fields is well known (Townsend *et al.*, 2002). Some substituted pyrimidines and their derivaties have been reported to possess anti-microbial and anti-fungal activities (El-Hashash *et al.*, 1993). Pyrimidines have incidental anti-viral activity against herpes and vaccinia infections (Calabresi *et al.*, 1975). A review on pyrimidines as anti-inflammatory agent is described by Amir *et al.* (2007). Sulfonamides are an important class of anti-bacterial drugs used in medicine and veterinary practice. Sulfa drugs are widely used in the treatment of infections, especially for patients intolerant to antibiotics. The vast commercial success of these medicinal agents has made the chemistry of sulfonamides to become a major area of research and an important branch of commercial importance in pharmaceutical sciences (Nagaraja *et al.*, 2003). In view of the importance of the title compound possessing potential anti-bacterial properties, its crystal structure is reported herein.

In the title sulfonamide compound (Fig. 1), the geometry around the S1 atom is a distorted tetrahedron, comprising of atoms O1 and O2 of the sulfonyl group, C6 atom of benzene ring and the amino atom N2. The O1–S1–O2 and O2–S1–N2 angles are 119.20 (5) and 102.20 (4)°, respectively, and the C6–S1–N2–C7 torsion angle is -68.95 (9)°. The pyrimidine ring is essentially planar, with r.m.s. deviation of -0.020 (1) Å, and is almost perpendicular to the benzene ring (C1-C6), as indicated by the dihedral angle of 87.57 (5)°. The bond lengths (Allen *et al.*, 1987) and angles are within normal ranges and are comparable to a related structure (Chohan *et al.*, 2008). In the crystal structure, pairs of intermolecular N2—H1N2···O2^iii^ hydrogen bonds (see Table 1 for symmetry code) generate *R*^2^_2_(8) ring motifs (Bernstein *et al.*, 1995). Neighbouring molecules are linked into a three-dimensional extended network by intermolecular N1—H1N1···N4, N1—H2N1···O1 and C4—H4A···O1 hydrogen bonds (Fig. 2).

### Experimental

The title compound was obtained as a gift sample from R. L. Fine Chem, Bangalore, India. The compound was used without further purification. Single crystals of good quality were obtained from slow evaporation of an acetonitrile solution. *M.p.* 447–450 K.

### Refinement

All the H atoms were located in a difference Fourier map and allowed to refine freely [range of C—H = 0.90 (2) - 0.991 (19) Å].

### Crystal data

**Table:**

C_11_H_11_ClN_4_O_3_S	*F*(000) = 648
*M**_r_* = 314.75	*D*_x_ = 1.539 Mg m^−^^3^
Monoclinic, *P*2_1_/*c*	Mo *K*α radiation, λ = 0.71073 Å
Hall symbol: -P 2ybc	Cell parameters from 9451 reflections
*a* = 12.8792 (6) Å	θ = 3.1–35.0°
*b* = 13.3557 (6) Å	µ = 0.45 mm^−^^1^
*c* = 8.0867 (4) Å	*T* = 100 K
β = 102.396 (1)°	Plate, colourless
*V* = 1358.57 (11) Å^3^	0.44 × 0.33 × 0.12 mm
*Z* = 4	

### Data collection

**Table:**

Bruker SMART APEX DUO area-detector diffractometer	4863 independent reflections
Radiation source: fine-focus sealed tube	4292 reflections with *I* > 2σ(*I*)
graphite	*R*_int_ = 0.026
φ and ω scans	θ_max_ = 32.5°, θ_min_ = 1.6°
Absorption correction: multi-scan (*SADABS*; Bruker, 2009)	*h* = −19→18
*T*_min_ = 0.829, *T*_max_ = 0.950	*k* = −20→20
20122 measured reflections	*l* = −11→12

### Refinement

**Table:**

Refinement on *F*^2^	Primary atom site location: structure-invariant direct methods
Least-squares matrix: full	Secondary atom site location: difference Fourier map
*R*[*F*^2^ > 2σ(*F*^2^)] = 0.030	Hydrogen site location: inferred from neighbouring sites
*wR*(*F*^2^) = 0.089	All H-atom parameters refined
*S* = 1.04	*w* = 1/[σ^2^(*F*_o_^2^) + (0.0475*P*)^2^ + 0.4528*P*] where *P* = (*F*_o_^2^ + 2*F*_c_^2^)/3
4863 reflections	(Δ/σ)_max_ < 0.001
225 parameters	Δρ_max_ = 0.53 e Å^−^^3^
0 restraints	Δρ_min_ = −0.37 e Å^−^^3^

### Special details

**Table:**

Experimental. The crystal was placed in the cold stream of an Oxford Cyrosystems Cobra open-flow nitrogen cryostat (Cosier & Glazer, 1986) operating at 100.0 (1)K.
Geometry. All esds (except the esd in the dihedral angle between two l.s. planes) are estimated using the full covariance matrix. The cell esds are taken into account individually in the estimation of esds in distances, angles and torsion angles; correlations between esds in cell parameters are only used when they are defined by crystal symmetry. An approximate (isotropic) treatment of cell esds is used for estimating esds involving l.s. planes.
Refinement. Refinement of F^2^ against ALL reflections. The weighted R-factor wR and goodness of fit S are based on F^2^, conventional R-factors R are based on F, with F set to zero for negative F^2^. The threshold expression of F^2^ > 2sigma(F^2^) is used only for calculating R-factors(gt) etc. and is not relevant to the choice of reflections for refinement. R-factors based on F^2^ are statistically about twice as large as those based on F, and R- factors based on ALL data will be even larger.

### Fractional atomic coordinates and isotropic or equivalent isotropic displacement parameters (Å^2^)

**Table:**

	*x*	*y*	*z*	*U*_iso_*/*U*_eq_	
Cl1	0.32221 (2)	0.130087 (19)	0.89383 (4)	0.02300 (7)	
S1	0.150384 (18)	0.591224 (17)	0.98106 (3)	0.01312 (6)	
O1	0.19845 (6)	0.64388 (6)	0.86316 (9)	0.01714 (14)	
O2	0.03978 (6)	0.60773 (6)	0.97922 (10)	0.01793 (14)	
O3	0.15020 (6)	0.26617 (6)	0.97198 (9)	0.01711 (14)	
N1	0.40417 (8)	0.62924 (7)	1.67597 (11)	0.02030 (18)	
N2	0.15327 (7)	0.46898 (6)	0.94120 (11)	0.01577 (15)	
N3	0.32756 (7)	0.46232 (7)	0.89578 (12)	0.01792 (16)	
N4	0.40819 (7)	0.30443 (7)	0.86117 (13)	0.01997 (17)	
C1	0.32974 (8)	0.63720 (7)	1.21150 (12)	0.01551 (17)	
C2	0.38928 (8)	0.64543 (8)	1.37493 (13)	0.01691 (17)	
C3	0.34401 (8)	0.62413 (7)	1.51452 (12)	0.01546 (17)	
C4	0.23638 (8)	0.59518 (8)	1.48550 (13)	0.01756 (18)	
C5	0.17708 (8)	0.58676 (8)	1.32262 (13)	0.01685 (18)	
C6	0.22376 (7)	0.60750 (7)	1.18497 (12)	0.01355 (16)	
C7	0.24116 (7)	0.41496 (7)	0.92115 (12)	0.01407 (16)	
C8	0.23504 (7)	0.30973 (7)	0.92579 (12)	0.01413 (16)	
C9	0.32214 (8)	0.25928 (7)	0.89322 (12)	0.01631 (17)	
C10	0.40614 (8)	0.40413 (8)	0.86664 (15)	0.0207 (2)	
C11	0.07329 (9)	0.21978 (10)	0.83669 (16)	0.0253 (2)	
H1A	0.3591 (13)	0.6546 (12)	1.120 (2)	0.025 (4)*	
H2A	0.4615 (13)	0.6672 (13)	1.393 (2)	0.027 (4)*	
H4A	0.2078 (13)	0.5806 (12)	1.581 (2)	0.027 (4)*	
H5A	0.1038 (13)	0.5624 (12)	1.303 (2)	0.026 (4)*	
H10A	0.4693 (14)	0.4379 (13)	0.847 (2)	0.030 (4)*	
H11A	0.0146 (14)	0.2042 (14)	0.883 (2)	0.035 (4)*	
H11B	0.0532 (14)	0.2688 (14)	0.743 (2)	0.038 (5)*	
H11C	0.0991 (15)	0.1654 (16)	0.795 (2)	0.043 (5)*	
H1N1	0.4646 (14)	0.6617 (14)	1.695 (2)	0.033 (4)*	
H2N1	0.3696 (15)	0.6271 (14)	1.759 (2)	0.034 (5)*	
H1N2	0.1012 (14)	0.4342 (13)	0.968 (2)	0.030 (4)*	

### Atomic displacement parameters (Å^2^)

**Table:**

	*U*^11^	*U*^22^	*U*^33^	*U*^12^	*U*^13^	*U*^23^
Cl1	0.02251 (13)	0.01320 (11)	0.03324 (15)	0.00115 (8)	0.00591 (10)	−0.00126 (9)
S1	0.01184 (10)	0.01368 (11)	0.01457 (11)	0.00149 (7)	0.00442 (8)	0.00027 (7)
O1	0.0191 (3)	0.0179 (3)	0.0156 (3)	0.0009 (3)	0.0064 (3)	0.0026 (2)
O2	0.0121 (3)	0.0195 (3)	0.0228 (3)	0.0032 (2)	0.0050 (3)	0.0005 (3)
O3	0.0161 (3)	0.0188 (3)	0.0177 (3)	−0.0056 (3)	0.0064 (3)	−0.0027 (3)
N1	0.0241 (4)	0.0228 (4)	0.0140 (4)	−0.0020 (3)	0.0041 (3)	−0.0002 (3)
N2	0.0129 (3)	0.0139 (3)	0.0220 (4)	−0.0005 (3)	0.0071 (3)	−0.0028 (3)
N3	0.0143 (4)	0.0154 (4)	0.0260 (4)	0.0000 (3)	0.0085 (3)	−0.0005 (3)
N4	0.0161 (4)	0.0172 (4)	0.0285 (4)	0.0019 (3)	0.0091 (3)	0.0003 (3)
C1	0.0155 (4)	0.0164 (4)	0.0160 (4)	−0.0008 (3)	0.0063 (3)	0.0003 (3)
C2	0.0159 (4)	0.0189 (4)	0.0167 (4)	−0.0015 (3)	0.0053 (3)	0.0000 (3)
C3	0.0191 (4)	0.0130 (4)	0.0148 (4)	0.0011 (3)	0.0049 (3)	−0.0002 (3)
C4	0.0202 (4)	0.0185 (4)	0.0161 (4)	−0.0004 (3)	0.0087 (3)	0.0009 (3)
C5	0.0157 (4)	0.0188 (4)	0.0178 (4)	−0.0007 (3)	0.0076 (3)	0.0008 (3)
C6	0.0135 (4)	0.0136 (4)	0.0145 (4)	0.0006 (3)	0.0049 (3)	0.0000 (3)
C7	0.0123 (4)	0.0149 (4)	0.0154 (4)	0.0003 (3)	0.0041 (3)	−0.0017 (3)
C8	0.0137 (4)	0.0148 (4)	0.0144 (4)	−0.0013 (3)	0.0041 (3)	−0.0010 (3)
C9	0.0167 (4)	0.0135 (4)	0.0189 (4)	0.0011 (3)	0.0042 (3)	−0.0011 (3)
C10	0.0153 (4)	0.0180 (4)	0.0313 (5)	0.0004 (3)	0.0103 (4)	−0.0002 (4)
C11	0.0196 (5)	0.0299 (6)	0.0268 (5)	−0.0086 (4)	0.0058 (4)	−0.0109 (4)

### Geometric parameters (Å, °)

**Table:**

Cl1—C9	1.7255 (10)	C1—C2	1.3826 (14)
S1—O1	1.4283 (7)	C1—C6	1.3932 (13)
S1—O2	1.4383 (7)	C1—H1A	0.931 (16)
S1—N2	1.6662 (9)	C2—C3	1.4063 (13)
S1—C6	1.7292 (10)	C2—H2A	0.955 (17)
O3—C8	1.3590 (11)	C3—C4	1.4094 (14)
O3—C11	1.4480 (13)	C4—C5	1.3781 (15)
N1—C3	1.3694 (13)	C4—H4A	0.941 (17)
N1—H1N1	0.875 (18)	C5—C6	1.4016 (13)
N1—H2N1	0.879 (19)	C5—H5A	0.979 (16)
N2—C7	1.3809 (12)	C7—C8	1.4085 (14)
N2—H1N2	0.880 (18)	C8—C9	1.3816 (13)
N3—C7	1.3336 (12)	C10—H10A	0.973 (17)
N3—C10	1.3364 (13)	C11—H11A	0.938 (18)
N4—C10	1.3328 (14)	C11—H11B	0.991 (19)
N4—C9	1.3351 (13)	C11—H11C	0.90 (2)
			
O1—S1—O2	119.20 (5)	C3—C4—H4A	117.7 (10)
O1—S1—N2	108.81 (4)	C4—C5—C6	119.92 (9)
O2—S1—N2	102.20 (4)	C4—C5—H5A	119.9 (10)
O1—S1—C6	110.38 (5)	C6—C5—H5A	120.1 (10)
O2—S1—C6	109.14 (5)	C1—C6—C5	120.47 (9)
N2—S1—C6	106.10 (5)	C1—C6—S1	119.96 (7)
C8—O3—C11	115.81 (8)	C5—C6—S1	119.52 (8)
C3—N1—H1N1	119.3 (12)	N3—C7—N2	120.16 (9)
C3—N1—H2N1	116.6 (12)	N3—C7—C8	122.05 (9)
H1N1—N1—H2N1	117.5 (17)	N2—C7—C8	117.78 (8)
C7—N2—S1	125.98 (7)	O3—C8—C9	125.25 (9)
C7—N2—H1N2	116.1 (11)	O3—C8—C7	119.17 (8)
S1—N2—H1N2	114.8 (11)	C9—C8—C7	115.41 (9)
C7—N3—C10	116.09 (9)	N4—C9—C8	123.96 (9)
C10—N4—C9	114.94 (9)	N4—C9—Cl1	116.89 (7)
C2—C1—C6	119.60 (9)	C8—C9—Cl1	119.15 (8)
C2—C1—H1A	120.2 (10)	N4—C10—N3	127.44 (10)
C6—C1—H1A	120.1 (10)	N4—C10—H10A	115.8 (10)
C1—C2—C3	120.71 (9)	N3—C10—H10A	116.8 (10)
C1—C2—H2A	119.7 (10)	O3—C11—H11A	105.7 (11)
C3—C2—H2A	119.6 (10)	O3—C11—H11B	108.5 (11)
N1—C3—C2	120.53 (9)	H11A—C11—H11B	110.6 (15)
N1—C3—C4	120.49 (9)	O3—C11—H11C	112.6 (12)
C2—C3—C4	118.96 (9)	H11A—C11—H11C	111.6 (17)
C5—C4—C3	120.34 (9)	H11B—C11—H11C	107.8 (16)
C5—C4—H4A	122.0 (10)		
			
O1—S1—N2—C7	49.81 (10)	C10—N3—C7—N2	−175.76 (10)
O2—S1—N2—C7	176.75 (8)	C10—N3—C7—C8	3.19 (15)
C6—S1—N2—C7	−68.95 (9)	S1—N2—C7—N3	−14.89 (14)
C6—C1—C2—C3	0.01 (15)	S1—N2—C7—C8	166.11 (7)
C1—C2—C3—N1	177.89 (10)	C11—O3—C8—C9	−78.05 (13)
C1—C2—C3—C4	−0.66 (15)	C11—O3—C8—C7	106.84 (11)
N1—C3—C4—C5	−177.74 (10)	N3—C7—C8—O3	172.23 (9)
C2—C3—C4—C5	0.80 (15)	N2—C7—C8—O3	−8.80 (13)
C3—C4—C5—C6	−0.30 (15)	N3—C7—C8—C9	−3.36 (14)
C2—C1—C6—C5	0.51 (15)	N2—C7—C8—C9	175.62 (9)
C2—C1—C6—S1	−176.93 (8)	C10—N4—C9—C8	1.60 (15)
C4—C5—C6—C1	−0.37 (15)	C10—N4—C9—Cl1	−178.19 (8)
C4—C5—C6—S1	177.08 (8)	O3—C8—C9—N4	−174.46 (10)
O1—S1—C6—C1	−20.24 (9)	C7—C8—C9—N4	0.82 (14)
O2—S1—C6—C1	−153.06 (8)	O3—C8—C9—Cl1	5.32 (14)
N2—S1—C6—C1	97.49 (8)	C7—C8—C9—Cl1	−179.40 (7)
O1—S1—C6—C5	162.29 (8)	C9—N4—C10—N3	−1.89 (18)
O2—S1—C6—C5	29.47 (9)	C7—N3—C10—N4	−0.47 (18)
N2—S1—C6—C5	−79.98 (9)		

### Hydrogen-bond geometry (Å, °)

**Table:**

*D*—H···*A*	*D*—H	H···*A*	*D*···*A*	*D*—H···*A*
N1—H1N1···N4^i^	0.875 (19)	2.616 (18)	3.4230 (14)	153.8 (15)
N1—H2N1···O1^ii^	0.882 (18)	2.533 (19)	3.3274 (13)	150.2 (15)
N2—H1N2···O2^iii^	0.880 (18)	2.031 (18)	2.8866 (12)	163.7 (16)
C4—H4A···O1^ii^	0.944 (16)	2.460 (16)	3.2603 (13)	142.5 (13)

Symmetry codes: (i) −*x*+1, *y*+1/2, −*z*+5/2; (ii) *x*, *y*, *z*+1; (iii) −*x*, −*y*+1, −*z*+2.
